# Variations in histopathological and molecular subtypes between primary breast carcinoma and associated liver metastatic lesions

**DOI:** 10.3389/fonc.2026.1625082

**Published:** 2026-08-05

**Authors:** Fang Guo, Gelin ZhangYang, Pengfei Xu, Liting Jin

**Affiliations:** 1 Department of Pathology, Hubei Provincial Hospital of Traditional Chinese Medicine, Wuhan, Hubei, China; 2 Department of Pathology, Hubei Cancer Hospital, Tongji Medical College, Huazhong University of Science and Technology, Wuhan, Hubei, China; 3 Department of Breast Cancer Center, Hubei Cancer Hospital, Tongji Medical College, Huazhong University of Science and Technology, National Key Clinical Specialty, Hubei Provincial Clinical Research Center for Breast Cancer, Wuhan Clinical Research Center for Breast Cancer, Wuhan, Hubei, China

**Keywords:** breast cancer, grade, liver metastasis, LPFS, subtype

## Abstract

**Introduction:**

Evidence suggests that histopathological features and molecular subtypes influence the prognosis of breast carcinoma; however, their roles in liver metastatic breast cancer remain unclear. This study aimed to compare histopathological characteristics and molecular subtypes between primary breast tumors and paired liver metastases, and to evaluate their prognostic significance.

**Methods:**

This retrospective study included 41 patients with liver metastatic breast cancer. Histopathological re-evaluation was performed for paired primary tumors and liver metastases, including tumor grade, lymphovascular invasion (LVI), lymph node (LN) status, and molecular subtype. Clinical data and liver progression-free survival (LPFS) were also analyzed. Patients were stratified according to LPFS ≤60 months and LPFS >60 months.

**Results:**

Patients with LPFS ≤60 months showed higher rates of LVI and positive LN metastasis in primary tumors than those with LPFS >60 months. Liver metastases from patients with shorter LPFS had a higher proportion of grade 3 tumors. Tumor grade increased in liver metastases compared with primary tumors, with grade 3 tumors accounting for 56.1% of metastases versus 22.0% of primary tumors. Overall, 39.0% of cases showed grade progression, whereas 24.4% showed molecular subtype changes. Molecular subtype composition remained largely unchanged between primary and metastatic lesions.

**Discussion:**

Histopathological discordance, particularly grade progression between primary tumors and liver metastases, may provide additional prognostic stratification beyond conventional baseline tumor characteristics. These findings highlight the importance of re-biopsy and dynamic pathological reassessment of metastatic lesions, which may directly influence therapeutic decision-making in metastatic breast cancer. Comprehensive pathological evaluation of liver metastases is recommended to guide prognosis assessment and treatment strategies.

## 1 Introduction

Breast cancer remains the leading cause of cancer-related mortality among women globally, according to the Global Cancer Statistics 2020 (1). In China, breast cancer was identified as the most prevalent malignancy among females, accounting for 16.7% (306,000) of all new cancer cases in 2016, according to a recent report published in Journal of the National Cancer Center in 2022 (2). Metastasis to distant organs, including the lungs, bones, brain, liver, and adrenal glands, is a leading cause of death in breast cancer and characterizes a systemic condition with a poor prognosis. Approximately 20–30% of patients diagnosed with early-stage breast cancer die from metastatic disease (3). The liver is among the most frequently affected metastatic sites for solid tumors and ranks as the third most common site for breast cancer metastasis (4). Patients with breast cancer liver metastases tend to be younger than those with metastases to the lungs or other extrahepatic sites. These patients exhibit a three-year overall survival (OS) rate of 38.2% and a three-year breast cancer-specific survival rate of 67.7% (5). Histologically, approximately 86.1% of these patients present with invasive ductal carcinoma; molecularly, they predominantly exhibit estrogen receptor (ER) and progesterone receptor (PR) positivity, along with HER2 negativity (6).

The interval between diagnosis and treatment of primary tumors and the first appearance of liver metastases varies among patients. It remains unclear whether patients classified according to different metastatic intervals correspond to distinct histopathological types or molecular phenotypes. Furthermore, while subtype discordance in receptor status (ER/PR and HER2) and tumor grade between primary and recurrent or metastatic breast cancer is not uncommon, the role of such changes in liver metastasis remains poorly understood.

Given this context, we aimed to evaluate the clinical and histopathological characteristics as well as molecular subtypes in both primary tumors and paired liver metastases of breast carcinoma. Additionally, we sought to analyze differences among patients with varying intervals to liver metastasis.

## 2 Methods

### 2.1 Patients

Forty-one patients diagnosed with primary breast cancer who subsequently developed liver metastases were evaluated and/or treated at the Breast Tumor Center of Hubei Cancer Hospital, Wuhan, China, between November 2006 and July 2023. All patients were initially diagnosed with stage M0 breast carcinoma (T1–T4, N0–N3) and received standard treatment, including neoadjuvant therapy or upfront surgery, followed by adjuvant therapies such as chemotherapy, radiotherapy, endocrine therapy, and/or anti-HER2 targeted therapy. Liver metastases were detected at variable intervals, ranging from several months to years after the initial diagnosis.

This study adopted a retrospective consecutive sampling strategy, whereby all eligible patients within the study period were included to minimize potential selection bias. Due to the relative rarity of cases with paired primary and liver metastatic pathological specimens, no formal sample size calculation was performed. Instead, the sample size was determined based on case availability, which is consistent with prior studies investigating matched primary–metastatic breast cancer cohorts.

The inclusion criteria were as follows: (1) histologically confirmed primary breast carcinoma; (2) pathologically confirmed liver metastasis with available paired tissue specimens; (3) complete clinicopathological and follow-up data; (4) no evidence of distant metastasis (M0) at initial diagnosis.

The exclusion criteria were: (1) incomplete clinical, pathological, or follow-up data; (2) history of other malignancies; (3) absence of available paired liver metastatic tissue; (4) poor-quality specimens unsuitable for histopathological or immunohistochemical reassessment.

Specimen quality for histopathological and IHC reassessment was judged unsuitable when tissue area was <100 tumor cells per core, or when necrosis/crush artifact precluded reliable grading; such cases were excluded under criterion (4) above.

### 2.1 Histopathological and immunohistochemical assessment

Tumor tissue specimens from primary breast carcinoma (obtained via surgical resection or core needle biopsy) and paired liver metastases (obtained via core needle biopsy or fine-needle aspiration) were retrospectively retrieved and re-evaluated. Histopathological parameters included tumor type, histological grade (according to the Nottingham grading system), lymphovascular invasion (LVI), and lymph node (LN) status.

All slides were independently reviewed by two experienced breast pathologists (>5 years of diagnostic experience) who were blinded to clinical outcomes. In cases of discrepancy, a consensus diagnosis was reached through joint review.

Immunohistochemical (IHC) staining was performed using an automated staining platform (Ventana Benchmark XT system, Roche Diagnostics, Basel, Switzerland) according to the manufacturer’s protocols. The primary antibodies used were as follows: estrogen receptor (ER; clone SP1, Roche Diagnostics), progesterone receptor (PR; clone 1E2, Roche Diagnostics), HER2 (clone 4B5, Roche Diagnostics), and Ki67 (clone MIB-1, Dako/Agilent Technologies). Appropriate positive and negative controls were included in each staining run to ensure assay reliability.

The evaluation of IHC markers was conducted according to the 5th edition of the WHO Classification of Breast Tumors and ASCO/CAP guidelines, as detailed below:

1. ER and PR status were considered positive when ≥1% of tumor cell nuclei showed immunoreactivity.
2. HER2 status was scored as 0, 1+, 2+, or 3+ based on membrane staining intensity and completeness. Scores of 0–1+ were considered negative, and 3+ was considered positive. Cases with equivocal HER2 expression (2+) were further analyzed using fluorescence *in situ* hybridization (FISH). HER2 amplification was defined as a HER2/CEP17 ratio ≥2.0 or an average HER2 copy number ≥6 signals per cell, according to ASCO/CAP guidelines.
3. Ki67 proliferation index was assessed by counting at least 500 tumor cells under high-power magnification (×400). In tumors exhibiting intratumoral heterogeneity, hotspot areas with the highest nuclear staining were selected, and 10 representative high-power fields were evaluated. In cases with homogeneous staining distribution, 10 randomly selected fields were assessed. The Ki67 index was expressed as the percentage of positively stained tumor nuclei, and categorized as low (<14%) or high (≥14%).
4. Based on IHC results, tumors were classified into five molecular subtypes:• Luminal A-like: ER and PR positive, HER2 negative, low Ki67 proliferation index;
  • Luminal A-like: ER and/or PR positive, HER2 negative, low Ki67;
  • Luminal B-like (HER2-negative): ER positive, HER2 negative, with high Ki67 or low/negative PR;
  • Luminal B-like (HER2-positive): ER positive with HER2 overexpression or amplification;
  • HER2-positive (non-luminal): HER2 positive, ER and PR negative;
  • Triple-negative: ER, PR, and HER2 all negative.

### 2.3 Analyze clinical and pathological characteristic

The interval from the date of initial diagnosis and treatment to the date of liver progression was defined as liver progression-free survival (LPFS). Follow-up ended at the patient’s last available clinical information or at the final follow-up on July 20, 2023. Patients were divided into two groups based on their LPFS for separate analyses: the first group included 27 women with LPFS ≤ 60 months (mean ± SD: 23.2 ± 16), and the second group comprised 14 women with LPFS > 60 months (mean ± SD: 113.4 ± 49.5). Variables analyzed included age, tumor location, tumor size, histopathological classification, grade, molecular subtype, lymphovascular invasion and lymph node status, number of liver metastases, treatments received, and presence of extrahepatic metastases.

The histopathological features of primary breast carcinomas and their matched liver metastases were compared, with particular focus on tumor grade and molecular subtype.

### 2.4 Statistical analysis

All statistical analyses were performed using SPSS software (version 20.0; IBM Corp., Armonk, NY, USA). Categorical variables were compared using the Chi-square test or Fisher’s exact test, as appropriate. Continuous variables were first assessed for normality using the Shapiro–Wilk test. Normally distributed variables were compared using the independent samples t-test, while non-normally distributed variables were analyzed using the Mann–Whitney U test.

Liver progression-free survival (LPFS) was defined as the interval from the initial diagnosis of primary breast cancer to the date of liver disease progression or last follow-up. Survival curves were estimated using the Kaplan–Meier method, and differences between groups were compared using the log-rank test.

To identify prognostic factors associated with LPFS, univariate Cox proportional hazards regression analysis was initially performed. Variables with p < 0.10 in univariate analysis were subsequently included in a multivariate Cox regression model to determine independent prognostic factors, adjusting for potential confounders including age, tumor stage, lymphovascular invasion (LVI), lymph node (LN) status, tumor grade, molecular subtype, and treatment modalities.

To address potential confounding, a multivariate Cox proportional hazards model was constructed including age at primary diagnosis (continuous), lymphovascular invasion (LVI, present vs. absent), lymph node metastasis (positive vs. negative), and primary tumor histological grade (grade 3 vs. grade 1–2) as covariates, based on clinical plausibility and univariate significance (P < 0.10). Given the modest sample size (n = 41), the number of covariates was restricted to maintain an adequate events-per-variable ratio. Since all enrolled patients had, by inclusion criteria, developed liver metastasis, LPFS was modeled as a fully observed (uncensored) time-to-event variable, consistent with the framework already used for the Kaplan–Meier and log-rank analyses described above.

Hazard ratios (HRs) and 95% confidence intervals (CIs) were calculated. All statistical tests were two-sided, and a p value < 0.05 was considered statistically significant.

## 3 Results

### 3.1 Pathological characteristics of primary breast carcinoma and clinical treatment

A total of 41 female patients with primary breast carcinoma and liver metastases were included in the analysis. Patient ages at initial diagnosis ranged from 28 to 60 years (mean ± SD, 45.6 ± 8.2). None presented with bilateral breast tumors. Approximately 73.2% of cases were classified as T3 stage, while 17.1% were lymph node-negative. All patients received either neoadjuvant therapy or underwent surgery directly, followed by postoperative treatment that included chemotherapy, radiotherapy, hormone therapy, or trastuzumab. Notably, 51.2% of patients had extrahepatic metastases involving the lungs, brain, and bones (see **Table 1**).

**Table 1:** Clinical characteristics of patients.

Variable	Patients, no.%, total (n=41)
Age*, y	
28-40	10 (24.4%)
41-50	20 (48.8%)
51-60	11 (26.8%)
Tumor location	
Left	21 (51.2%)
Right	20 (48.8%)
Bilateral	0
Pathological stage	
T1	5 (12.2%)
T2	30 (73.2%)
T3	3 (7.3%)
T4	3 (7.3%)
N0	7 (17.1%)
N1	14 (34.1%)
N2	10 (24.4%)
N3	10 (24.4%)
Chemotherapy	
Yes	39 (95.1%)
No	2 (4.9%)
Endocrine therapy	
Yes	24 (58.5%)
No	17 (41.5%)
Radiotherapy	
Yes	21 (51.2%)
No	20 (48.8%)
Extrahepatic metastatic sites to lung, brain and bone	
Yes	21 (51.2%)
No	20 (48.8%)

*The age when patient had primary breast carcinoma and first diagnosed.

### 3.2 Clinical and pathological characteristics in patients with different LPFS

For the analysis of clinical and pathological characteristics, patients were divided into two groups based on liver progression-free survival (LPFS). The short LPFS group (D1) included patients with LPFS ≤ 60 months (mean ± SD: 23.2 ± 16), whereas the long LPFS group (D2) comprised patients with LPFS > 60 months (mean ± SD: 113.4 ± 49.5). In primary breast carcinoma, the rates of lymphovascular invasion (LVI) and lymph node metastases were significantly higher in group D1 compared to group D2 (p < 0.05). No statistically significant differences were observed between the groups for other characteristics, including tumor location, T stage, grade, and molecular subtype. Regarding liver metastases, a significant difference in histopathological grade was identified between groups D1 and D2 (p < 0.05). Specifically, in group D1, 8 of 27 patients (29.6%) exhibited histopathological grade 2, whereas in group D2, 10 of 14 patients (71.4%) showed grade 2. Conversely, 19 of 27 patients (70.4%) in group D1 had grade 3 tumors, compared to 4 of 14 patients (28.6%) in group D2 (see **Table 2**).

**Table 2:** Clinical and pathological characteristics of patients with different LPFS.

Variable		Groups		*p* value
		LPFS ≤ 60 months	LPFS > 60 months	
Patients, No. (n=41)		27	14	/
Patient’s age* (y)		46.5 ± 8.1	44 ± 8.7	0.367
Primary breast carcinoma location				
left		16 (59.3%)	5 (35.7%)	0.271
right		11 (40.7%)	9 (64.3%)
LVI		22 (81.5%)	6 (42.9%)	0.017
Metastases of LN		25 (92.6%)	9 (64.3%)	0.035
T stage				
T1	1	3 (11.1%)	2 (14.3%)	0.993
T2	2	20 (74.1%)	10 (71.4%)	
T3	3	2 (7.4%)	1 (7.1%)	
T4	4	2 (7.4%)	1 (7.1%)	
Grade				
G1	1	1 (3.7%)	2 (14.3%)	0.463
G2	2	20 (74.1%)	9 (64.3%)	
G3	3	6 (22.2%)	3 (21.4%)	
Molecular subtype				
A		2 (7.4%)	2 (14.3%)	0.525
B		15 (55.6%)	6 (42.9%)	
C		3 (11.1%)	4 (28.6%)	
D		5 (18.5%)	1 (7.1%)	
E		2 (7.4%)	1 (7.1%)	
Liver metastases				
Number				
<3		5 (18.5%)	1 (7.1%)	0.609
≥3		22 (81.5%)	13 (92.9%)	
Size (CM)		3.3 ± 1.7	2.8 ± 1.5	0.432
Grade				
1		0	0 (0%)	0.026
2		8 (29.6%)	10 (71.4%)	
3		19 (70.4%)	4 (28.6%)	
Molecular subtype				
A		1 (3.7%)	1 (7.1%)	0.975
B		11 (40.7%)	6 (42.9%)	
C		3 (11.1%)	2 (14.3%)	
D		7 (25.9%)	3 (21.4%)	
E		5 (18.5%)	2 (14.3%)	
Chemotherapy		26 (96.3%)	13 (92.9%)	1
Endocrine therapy		14 (51.9%)	3 (21.4%)	0.123
Radiotherapy		12 (44.4%)	8 (57.1%)	0.659
Coexistence of extrahepatic metastases		11 (40.7%)	10 (71.4%)	0.125

*The age when patient had primary breast carcinoma and first diagnosed;.

A, Luminal A-like; B, Luminal B-like (HER2-negative); C, Luminal B-like (HER2-positive); D, HER2-positive (non-luminal); E, Triple-negative.

### 3.3 Progression of histopathological grade and alteration of molecular subtype between primary and metastatic tumor

The differences in histopathological grade between primary breast carcinoma and liver metastases were statistically significant (p < 0.05). In primary breast carcinoma specimens, the majority of cases (29 of 41, 70.7%) were classified as grade 2. In contrast, liver metastases showed a reduction in grade 2 cases (18 of 41, 43.9%) and an increase in grade 3 cases, which accounted for more than half (23 of 41, 56.1%). No statistically significant differences were observed in molecular subtypes between primary tumors and liver metastases (p > 0.05) (**Table 3**).

**Table 3:** Comparison of histopathological grade and molecular subtype between primary breast carcinomas and paired liver metastases.

Variable	Patients, no.%, total (n=41)		p value
	Breast carcinoma	Liver metastases	
Histopathological grade			
1	3 (7.3%)	0 (0%)	0.003
2	29 (70.7%)	18 (43.9%)	
3	9 (22%)	23 (56.1%)	
Molecular subtype (patients, No.%)			
A	4 (9.8%)	2 (4.9%)	0.403
B	21 (51.2%)	17 (41.5%)	
C	7 (17.1%)	5 (12.2%)	
D	6 (14.6%)	10 (24.4%)	
E	3 (7.3%)	7 (17.1%)	

A, Luminal A-like; B, Luminal B-like (HER2-negative); C, Luminal B-like (HER2-positive); D, HER2-positive (non-luminal); E, Triple-negative.

**Figure 1:**
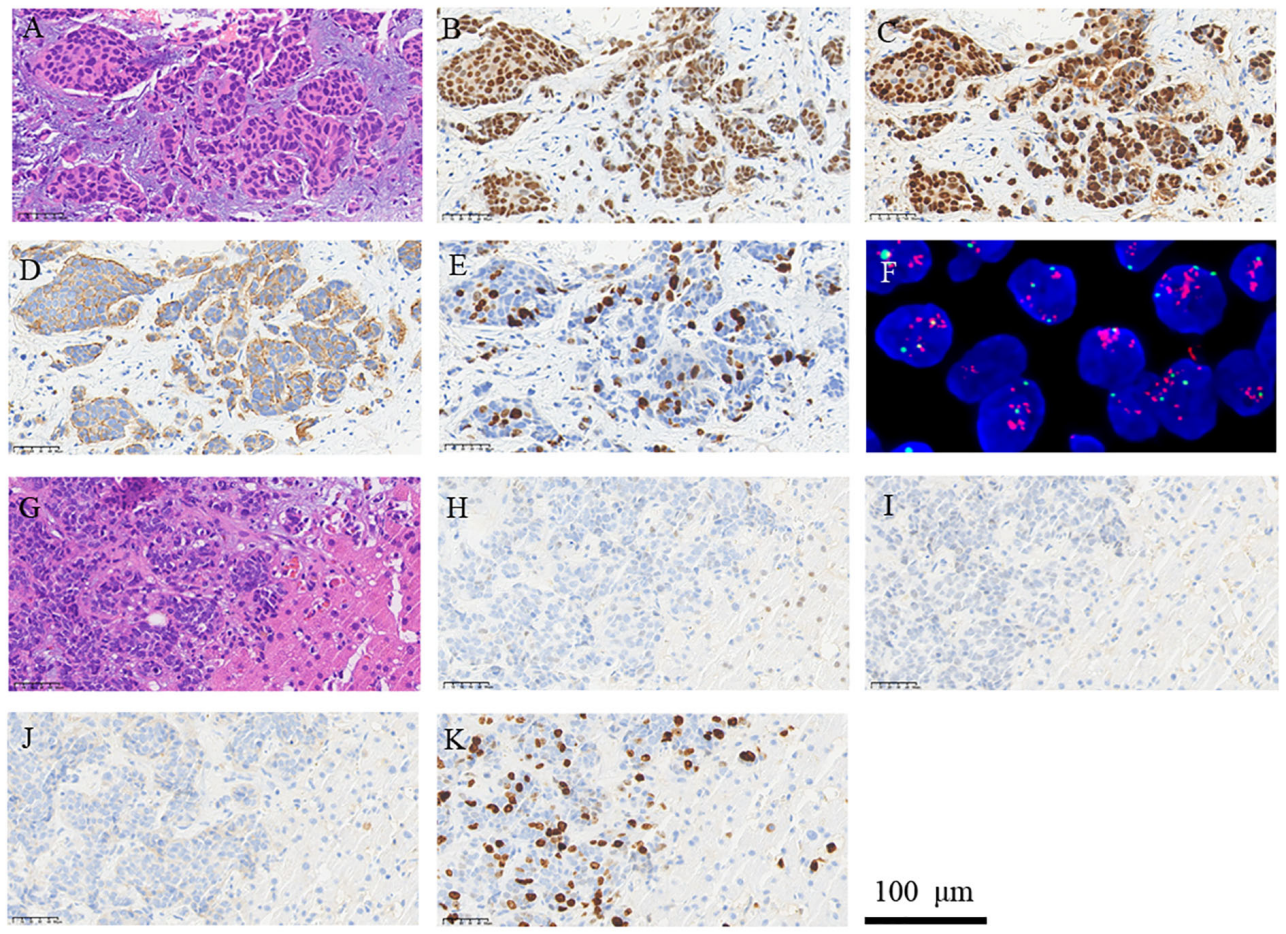
Illustrates the molecular subtype differences between the primary breast carcinoma and matched liver metastases in patient 39, demonstrating a transition from a luminal B-like (HER2-positive) phenotype to a triple-negative profile. Panels **(A–F)** correspond to the primary breast carcinoma, while panels **(G–K)** show the liver metastases. Invasive breast carcinoma of no special type was composed of tumor cells arranged in cords, clusters, and trabeculae infiltrating the desmoplastic fibrous stroma **(A)** ; hematoxylin and eosin stain). Immunohistochemical staining revealed strong nuclear expression of estrogen receptor (ER) **(B)** and progesterone receptor (PR) **(C)**, with HER2 showing an equivocal IHC score of 2+ **(D)**. HER2 overexpression was confirmed by fluorescence *in situ* hybridization (FISH) **(F)**. The Ki67 proliferation index was approximately 20% [**(E)**; immunohistochemical stain]. The metastatic tumor cells in the liver [panel **(G)**] displayed a similar histopathological morphology to the primary tumor [left side of **(G)**]. These tumor cells replaced normal hepatocytes while preserving the liver architecture and co-opting sinusoidal blood vessels [right side of **(G)**]. Notably, ER **(H)**, PR **(I)**, and HER2 **(J)** were negative in the metastatic lesions, while the Ki67 proliferation index increased to approximately 40% (K; Ki67 immunohistochemical stain). The scale bar indicates 100 μm.

Further paired analysis showed that histopathological grade progression was observed in 16 of 41 patients: two progressed from grade 1 to grade 2, one from grade 1 to grade 3, and thirteen from grade 2 to grade 3 (**Table 4**). In addition, subtype conversion occurred in 10 of the 41 patients. Specifically, one patient shifted from luminal A-like to luminal B-like (HER2-negative), one from luminal A-like to HER2-positive (non-luminal), one from luminal B-like (HER2-negative) to luminal B-like (HER2-positive), four from luminal B-like (HER2-negative) to triple-negative, two from luminal B-like (HER2-positive) to HER2-positive (non-luminal), and one from luminal B-like (HER2-positive) to triple-negative status (**Figure 1**; **Table 4**).

**Table 4:** Concordance and discordance of histopathological grade and molecular subtype between primary breast carcinomas and paired liver metastases.

Variable	Patients, Total (n=41)	Liver metastases							
		Grade			Subtype				
		1	2	3	A	B	C	D	E
Breast carcinoma									
Histopathological grade									
1	3	0	2	1					
2	29	0	16	13					
3	9	0	0	9					
Molecular subtype									
A	4				2	1	0	1	0
B	21				0	16	1	0	4
C	7				0	0	4	2	1
D	6				0	0	0	6	0
E	3				0	0	0	0	3

A, Luminal A-like; B, Luminal B-like (HER2-negative); C, Luminal B-like (HER2-positive); D, HER2-positive (non-luminal); E, Triple-negative.

### 3.4 Multivariate Cox regression analysis of factors associated with LPFS

To identify independent predictors of LPFS and control for potential confounding among LVI, LN status, tumor grade and age, univariate and multivariate Cox proportional hazards regression were performed (**Table 5**). In univariate analysis, LVI was significantly associated with shorter LPFS (HR 2.60, 95% CI 1.19–5.67, P = 0.016), while age, LN metastasis and tumor grade were not statistically significant. After adjusting for age, LN status and grade in the multivariate model, the association between LVI and LPFS was attenuated and no longer reached statistical significance (HR 2.40, 95% CI 0.90–6.40, P = 0.082), although the point estimate remained consistent in direction and magnitude with the univariate result. None of age, LN status, or tumor grade emerged as independent predictors of LPFS after mutual adjustment (all P > 0.28). These findings suggest that the prognostic effect of LVI observed in univariate analysis is at least partly explained by its correlation with lymph node involvement, and that no single baseline pathological feature independently and robustly predicts LPFS in this cohort — reinforcing the rationale for composite, dynamic pathological reassessment rather than reliance on any single baseline marker.

**Table 5:** Univariate and multivariate Cox regression analysis of factors associated with LPFS (n = 41).

Variable	Category	Univariate HR (95% CI)	P	Multivariate HR (95% CI)	P
Age	per 1-year increase	1.03 (0.98–1.07)	0.230	1.02 (0.98–1.07)	0.319
LVI	present vs. absent	2.60 (1.19–5.67)	0.016	2.40 (0.90–6.40)	0.082
LN metastasis	positive vs. negative	1.79 (0.78–4.12)	0.168	1.24 (0.42–3.60)	0.698
Primary tumor grade	grade 3 vs. grade 1–2	1.46 (0.68–3.14)	0.330	1.55 (0.70–3.43)	0.284

## 4 Discussion

Primary breast cancer has been extensively studied, particularly regarding hormone receptor status, HER2 overexpression, histological grade, and nodal status, which are established prognostic indicators (7–10). Among molecular subtypes, luminal A exhibits the highest survival rates, followed by luminal B-like HER2-negative and luminal B-like HER2-positive subtypes, while HER2-enriched and triple-negative breast cancers (TNBC) show the poorest prognoses (11). In this study, nearly half of the primary breast carcinoma cases (21/41, 51.2%) were classified as luminal B-like HER2-negative, suggesting a higher predisposition of this subtype for liver metastasis.

Unlike conventional studies focusing solely on primary tumor characteristics, this study emphasizes the dynamic evolution of tumor biology during liver metastasis, particularly histological grade progression and molecular subtype conversion. These findings suggest that static evaluation at initial diagnosis may be insufficient, and longitudinal pathological assessment may offer incremental prognostic and therapeutic value.

Histopathological grading remains an important prognostic factor, with higher tumor grades correlating with worse survival outcomes (12). Our cohort primarily consisted of grade 2 tumors (29/41, 70.7%) and grade 3 tumors (9/41, 22%), with few grade 1 cases (3/41, 7.3%). However, tumor grade distribution did not differ significantly between groups with long and short liver progression-free survival (LPFS), indicating that tumor grade alone may not predict LPFS but may influence liver metastasis progression (23).

Lymphovascular invasion (LVI) in the primary tumor emerged as a significant predictor of metastasis development and overall survival. Prior studies have demonstrated that LVI positivity is associated with poorer survival outcomes—even in early-stage breast cancer—compared to LVI-negative cases (13). Our subgroup analysis corroborates these findings, showing a higher frequency of LVI among patients with short LPFS (≤60 months) versus those with longer LPFS (>60 months). Other variables such as age, tumor location, T stage, primary tumor molecular subtype, chemotherapy, endocrine therapy, radiotherapy, and presence of extrahepatic metastasis did not show significant differences according to LPFS groups. Notably, in multivariate analysis adjusting for age, LN status and tumor grade, the effect of LVI on LPFS was attenuated to a non-significant trend (HR 2.40, 95% CI 0.90–6.40, P = 0.082), indicating that LVI’s prognostic value in univariate analysis may partly reflect its co-occurrence with LN metastasis rather than acting as a fully independent risk factor; this should be verified in larger, multicenter cohorts.

Recent research on breast cancer metastasis has focused on regional lymph node (LN) involvement (14), distant spread to organs like lung, brain, and bone (5, 15), molecular mechanisms of metastasis (16), heterogeneity among metastatic lesions (17), organ-specific metastatic patterns (18), and discordance of hormone receptor or HER2 status between primary and metastatic tumors (19). LN metastasis is a well-recognized risk factor negatively impacting overall and disease-free survival (14). Consistently, our data indicate that patients with positive LN metastasis face increased risk of liver metastasis and shorter LPFS. This association may partially relate to the growth patterns of metastatic tumor cells in the liver, notably replacement, desmoplastic, and pushing patterns (20), with the replacement pattern being predominant (92.7%; 38/41 cases) in our cohort. Most patients with positive LVI or LN metastasis exhibited this growth pattern (data not shown).

The presence of positive LVI and LN metastasis likely facilitates tumor cell entry into hepatic sinusoids via disruption of small vessel walls. Tumor cells then migrate within sinusoids, replacing normal hepatocytes. Interestingly, tumor cells may replace hepatocytes without triggering inflammation or fibrosis, preserving the reticulin framework and blood supply of liver parenchyma. This promotes tumor growth and contributes to shortened LPFS.

While histopathological grade of primary tumors is well-studied, data on metastatic tumor grading and its comparison with paired primaries are limited. In our study, 71.4% of patients with LPFS > 60 months had liver metastases of grade 2, whereas 70.4% with LPFS ≤ 60 months had grade 3 liver metastases. Moreover, 16 cases exhibited progression in histological grade between primary and metastatic lesions, underscoring the importance of grade escalation in liver metastasis progression.

Molecular subtype and receptor status discordances between primary breast carcinoma and metastases have also been documented (21). In our cohort, 10 cases showed alterations in ER/PR and HER2 status that led to reclassification of molecular subtype in metastatic lesions.

From a clinical standpoint, treatment modalities for breast cancer liver metastases include surgery, stereotactic body radiation therapy, and thermal ablation (radiofrequency or microwave) (6). Among these, curative hepatic resection offers the best chance for improved long-term survival (22).

## 5 Conclusions

In conclusion, both primary breast tumor features and corresponding liver metastases are critical to disease progression, but their prognostic contributions are not simply additive. In univariate analysis, lymphovascular invasion (LVI) and positive lymph node (LN) metastasis in the primary tumor were each associated with shorter LPFS, whereas histological grade and molecular subtype showed no significant baseline association. However, multivariate Cox regression adjusting for age, LN status, and tumor grade attenuated the effect of LVI to a non-significant trend (HR 2.40, 95% CI 0.90–6.40, P = 0.082), and no baseline pathological feature remained an independent, robust predictor of LPFS after mutual adjustment. This indicates that the prognostic value of LVI observed in univariate analysis is at least partly explained by its correlation with LN involvement, and that static baseline assessment alone is insufficient for risk stratification. Instead, the additive prognostic value lies in the dynamic evolution of tumor biology: 39% of cases showed histological grade progression and 24.4% showed molecular subtype conversion — including ER/PR and HER2 status alterations — between the primary tumor and liver metastasis, changes not captured by baseline evaluation alone. We therefore recommend that comprehensive baseline assessment of LVI, LN status, histological grade, and molecular subtype in the primary tumor be complemented by re-biopsy and dynamic pathological reassessment of liver metastases, to better inform individualized treatment decisions in metastatic breast cancer. Given the modest sample size (n = 41) of this single-center retrospective cohort, these findings — particularly the multivariate estimates — should be regarded as exploratory and warrant validation in larger, multicenter studies.

## Data Availability

The raw data supporting the conclusions of this article will be made available by the authors, without undue reservation.
